# Identification of the Thyrotropin-Releasing Hormone (TRH) as a Novel Biomarker in the Prognosis for Acute Myeloid Leukemia

**DOI:** 10.3390/biom12101359

**Published:** 2022-09-23

**Authors:** Yan Gao, Jia-Fan Zhou, Jia-Ying Mao, Lu Jiang, Xue-Ping Li

**Affiliations:** 1 Department of Medical Oncology, Sun Yat-sen University Cancer Center, Guangzhou 510060, China; 2 State Key Laboratory of Oncology in South China, Collaborative Innovation Center for Cancer Medicine, Guangzhou 510060, China; 3 Department of Nephrology, The Sixth Affiliated Hospital, Sun Yat-sen University, Guangzhou 510655, China; 4 Shanghai Institute of Hematology, State Key Laboratory of Medical Genomics, National Research Center for Translational Medicine at Shanghai, Ruijin Hospital, Shanghai Jiao Tong University School of Medicine, Shanghai 200025, China; 5 Department of Hematologic Oncology, Sun Yat-sen University Cancer Center, Guangzhou 510060, China

**Keywords:** acute myeloid leukemia, biomarkers, *TRH* expression, prognosis

## Abstract

Acute myeloid leukemia (AML) is a biologically and genetically heterogeneous hematological malignance with an unsatisfactory risk stratification system. Recently, through the novel single-cell RNA sequencing technology, we revealed heterogeneous leukemia myeloblasts in *RUNX1-RUNX1T1* AML. Thyrotropin-releasing hormone (*TRH*), as biomarkers of CD34^+^CD117^bri^ myeloblasts, were found to be prognostic in *RUNX1-RUNX1T1* AML. However, the clinical and genetic features of *TRH* in AML patients are poorly understood. Here, with data from TCGA AML, *TRH* was found to be downregulated in patients older than 60 years old, with *DNMT3A* and *NPM1* mutations, while overexpressed in patients with *KIT* mutations. This was further validated in three other cohorts of primary AML including Beat AML (*n* = 223), GSE6891 (*n* = 461), and GSE17855 (*n* = 237). Furthermore, we demonstrated that the expression of *TRH* in AML could be used to improve the ELN 2017 risk stratification system. In conclusion, our preliminary analysis revealed that *TRH*, a novel biomarker for AML patients, could be used to evaluate the survival of AML.

## 1. Introduction

Acute myeloid leukemia (AML) is a genetically and biologically heterogeneous hematological malignance with impaired hematopoiesis and bone marrow failure [1]. To induce a complete remission (CR), the backbone of intensive chemotherapy is still a combination of cytarabine and anthracyclines, including doxorubicin and daunorubicin, which reached a more than 65% CR rate in AML patients under 65 years old. However, relapse remains the leading cause of treatment failure [2,3]. It is estimated that the median overall survival (OS) of AML is no more than 8.5 months, and 5-year OS is only 24.0% [4].

Clinical risk stratification for AML is complex due to patient-associated factors and disease-related factors [3]. Especially for disease-related factors, the evaluation of leukemic cell genetic changes is still under exploration. Recently, through whole-exome sequencing (WES) and whole-genome sequencing (WGS), the Cancer Genome Atlas (TCGA) identified potential driver mutations and generated the genomic landscape of AML [5]. Moreover, the European Leukemia Net (ELN) guidelines incorporated gene mutations including *RUNX1* and *TP53* for AML stratification, which underlined the importance of genetic mutations in disease prognosis [6]. Gene expression based on RNA-seq was also demonstrated to be efficient in the risk stratification of AML [7].

Recently, we reported the heterogeneous populations among the CD34^+^ myeloblasts, namely, CD34^+^CD117^dim^ and CD34^+^CD117^bri^, in t (8;21) AML (*RUNX1-RUNX1T1* AML) [8]. Thyrotropin-releasing hormone (*TRH*), one of the biomarkers for CD34^+^CD117^bri^ myeloblasts, was observed to be highly expressed in AML patients, especially in those with *RUNX1-RUNX1T1* AML [9]. *TRH*, encoding a member of the thyrotropin-releasing hormone family, is involved in the hypothalamus–pituitary–thyroid (HPT) axis which exhibits feedback of thyroid hormone, thereby regulating metabolic and immunological homeostasis [10,11]. Research of the signaling pathways involving this gene might help provide therapeutic targets for metabolic disorders [12,13]. Our previous study also demonstrated that *TRH* expression was an independent prognostic factor for overall survival (OS) in t (8;21) AML after adjustment for clinical and genetic parameters [9]. Despite its importance in metabolism and immunity, as well as its potential prognostic value in t (8;21) AML, the relationship between *TRH* expression and clinical characteristics, genetic features, and immunological profiles in AML is poorly understood. In this work, we investigated the distribution of *TRH* expression in cancers, especially in different subtypes of AML. Based on our previous findings in t (8;21) AML, we further explored the clinical, genetic, immunological, and metabolic relevance, as well as molecular regulation correlated with *TRH* expression in AML patients. Through investigating the relationships between *TRH* expression and clinical prognosis, and genetic alterations, new ideas for the risk stratification and treatment of AML may be obtained.

## 2. Methods

### 2.1. Data Collection and Analysis

The transcriptome profiles of AML data, along with the detailed clinical information, were downloaded from public databases, including the Cancer Genome Atlas (TCGA) (https://portal.gdc.cancer.gov/ (accessed on 16 June 2021)) [5], Beat AML (www.vizome.org/aml (accessed on 15 January 2022)) [14], GSE6891 (https://www.ncbi.nlm.nih.gov/geo/ (accessed on 13 July 2022)) [15,16], and GSE17855 (https://www.ncbi.nlm.nih.gov/geo/ (accessed on 13 July 2022)) [17,18,19]. The transcriptomic data and detailed clinical information of patients with *RUNX1-RUNX1T1* AML patients were obtained from our previous studies [8,9].

### 2.2. Analysis of Gene Expression and Activated Pathways in RUNX1-RUNX1T1 AML

Differentiation expression analysis was performed by using DESeq2 [20]. The expression level of genes was evaluated by fragments per kilobase million (FPKM). Salmon [21] was used to determine and quantify the *RUNX1-RUNX1T1* transcript in t (8;21) AML as previously described [8]. Gene set enrichment analysis (GSEA) was performed with the GSEA software using the Kyoto Encyclopedia of Genes and Genomes (KEGG) gene set as described before [8,9].

### 2.3. Immune Infiltration Analysis

CIBERSORT was used to explore the proportion of immune cells in the bone marrow microenvironment as previously described [22]. Specifically, CIBERSORT was performed using the LM22 signature gene file with 100 permutations as the minimum. For each sample, a value was generated and only when *p* < 0.05 was viewed as significant and further analyzed. Correlation between *TRH* expression and immune cells inferred by CIBERSORT was determined by Spearman correlation.

### 2.4. GEPIA2, BloodSpot, Cistrome, cBioPortal, and GDSC Platforms

The online platform Gene Expression Profiling Interactive Analysis (GEPIA2, http://gepia2.cancer-pku.cn/ (accessed on 2 July 2022) [23] was used to compare the expression of *TRH* from TCGA and Genotype-Tissue Expression (GTEx) databases. BloodSpot (https://servers.binf.ku.dk/bloodspot/ (accessed on 15 September 2022)) [24] was used to investigate *TRH* expression with data from the collaborative Microarray Innovations in Leukemia (MILE) study [15,25]. The CistromeDB Toolkit (http://dbtoolkit.cistrome.org/ (accessed on 23 July 2022)) [26] and cBioPortal (http://cbioportal.org/ (accessed on 23 July 2022)) [27] were used to analyze published ChIP-seq data and to explore the association between the *TRH* expression and methylation levels, respectively. The expression level of *TRH* and predicted chemothearpeutic response were downloaded from the Genomics of Drug Sensitivity in Cancer (GDSC, https://www.cancerrxgene.org/ (accessed on 19 June 2022)) [28] to analyze the half-maximal inhibitory concentration (IC50) for doxorubicin, cytarabine, and all-trans retinoic acid (ATRA).

### 2.5. Statistical Analysis

The Kaplan–Meier method was used to estimate the probability of overall survival (OS). Log-rank tests were used to compare the *P* value. Statistical analyses were performed with R software (version 4.0.2, https://www.r-project.org/ (accessed on 20 August 2020)).

## 3. Results

### 3.1. Pan-Cancer Analysis Revealed the Unique TRH Expression Pattern in AML

**Figure 1:**
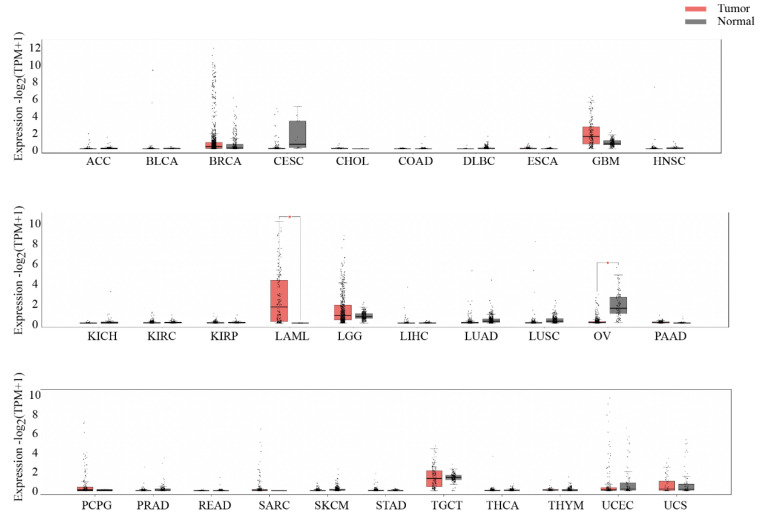
**Expression of *TRH* in pan-cancer and matched normal tissues**. The red bar represents tumor samples and the grey bar represents normal samples. *Y* axis, expression level of *TRH* based on the RNA-seq data. *X* axis, different types of cancers and matched normal data. AML refers to all subtypes in the TCGA project.

The expression of *TRH* in pan-cancer is rarely reported. Here, GEPIA2 [23] was utilized to analyze the RNA-seq data of pan-cancers from TCGA and the GTEx projects. We found that the expression level of *TRH* was actually low in most cancers and matched normal tissues (Figure 1). For hematological diseases, the expression levels of *TRH* in patients with t (8;21), inv (16), and t (15;17) were higher than those in patients with acute lymphoblastic leukemia (ALL), chronic lymphocytic leukemia (CLL), chronic myeloid leukemia (CML), and myelodysplastic syndrome (MDS) (Figure S1a). With single-cell RNA-seq data from Human Cell Landscape database [29], we found that the expression of *TRH* was rather low in most tissues (Figure S1b). In addition to the high expression level of *TRH* in AML, only ovarian serous cystadenocarcinoma (OV) had a significantly differential expression of *TRH* compared with the normal ovaries (*p* < 0.05) (Figure 1). Further survival analysis of *TRH* expression in the OV showed no prognostic significance, both for disease-free survival (*p* = 0.07) and overall survival (*p* = 0.23) (Figure S2a,b). Thus, the expression and clinical analysis in pan-cancers showed that *TRH* was a unique biomarker for AML.

Given that AML patients had a higher expression of *TRH* compared to healthy populations (Figure 1), and the Kaplan–Meier analysis of the *TRH*-low group indicated an inferior outcome for AML patients [9], we further explored the prognostic value of *TRH* expression in different AML FAB subgroups. For FAB-M2 and M4 AML patients, the *TRH*-high group presented a significantly better outcome than the *TRH*-low group (Figure S2e,f). For M0 and M1 AML patients, we observed a trend of better prognosis for the *TRH*-high group, though not reaching a statistically significance (*p* > 0.05) (Figure S2c,d). For the other M3, M5, M6, and M7 subtypes, the survival comparison was unable to be performed due to no comparable patients in two subgroups.

### 3.2. Metabolism Pathway Activation with TRH Expression in t (8; 21) AML

**Figure 2:**
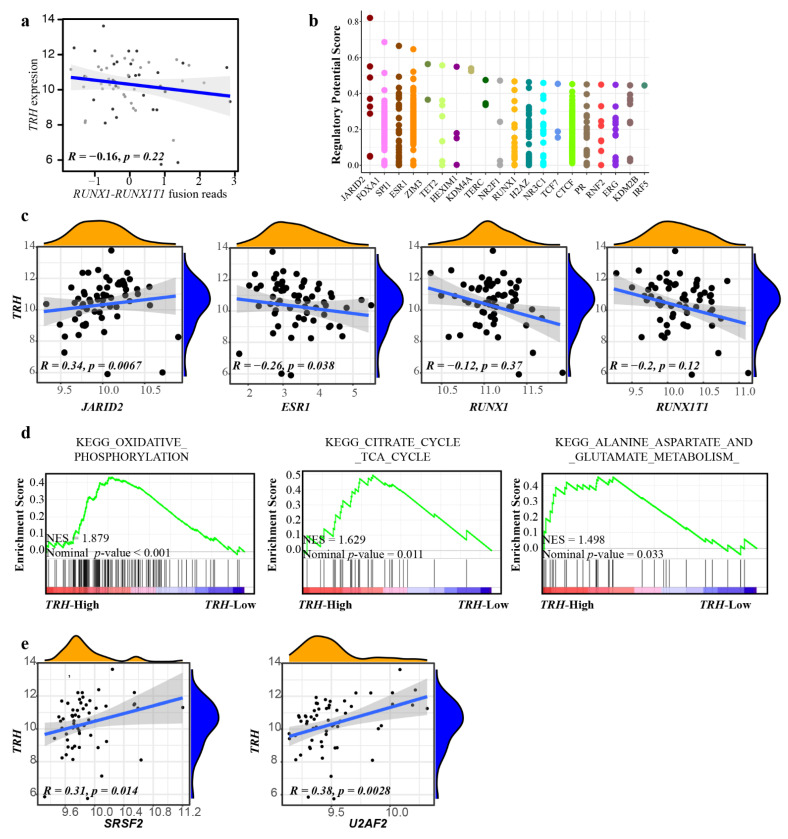
**Regulation network analysis of *TRH* expression in *RUNX1-RUNX1T1* AML patients**. (**a**) Correlation analysis of *TRH* expression with *RUNX1-RUNX1T1* fusion transcript expression based on the RNA-seq data of *RUNX1-RUNX1T1* AML patients. Spearman’s correlation analysis and correlation coefficient (*R*) are shown. (**b**) Top 20 putative and potential transcription factors for *TRH* from ChIP-seq dataset based on the Cistrome. *Y* axis, the regulatory potential score. *X* axis, different factors. Each dot represents a ChIP-seq sample. (**c**) Correlation analysis of *TRH* expression with transcription factors in *RUNX1-RUNX1T1* AML patients. Spearman’s correlation analysis and correlation coefficient (*R*) are shown. (**d**) Representative GSEA results of the *TRH*-high group in *RUNX1-RUNX1T1* AML patients. Normalized enriched score (NES) and nominal *p*-values are shown. (**e**) Correlation analysis of *TRH* expression with splicing factors in *RUNX1-RUNX1T1* AML. Spearman’s correlation analysis and correlation coefficient (*R*) are shown.

To analyze the regulation of *TRH* expression, the RNA-seq data from *RUNX1-RUNX1T1* AML patients (*n* = 62) [9] were analyzed and grouped into *TRH*-high (*n* = 31) and *TRH*-low (*n* = 31), based on the median level of *TRH* expression. Considering the role of the fusion *RUNX1-RUNX1T1* in t (8;21) AML, we analyzed and compared the *RUNX1-RUNX1T1* fusion between these groups (Figure 2a). We found that there was no significant correlation between the RNA expression of *RUNX1-RUNX1T1* fusion transcript and that of *TRH* (*R* = −0.16, *p* = 0.22). The Cistrome [26] database was used to predict the regulatory potential score of the top 20 transcription factors for *TRH*, which consisted of *JARID2*, *FOXA1*, *SPI1,* and *ESR1* (Figure 2b). Furthermore, using the RNA-seq data of *RUNX1-RUNX1T1* AML, we observed that there was a significant correlation between *TRH* expression and *JARID2* (*R* = 0.34, *p* = 0.0067), *ESR1* (*R* = −0.26, *p* = 0.038) (Figure 2c). However, there was no significant correlation between *TRH* expression and *RUNX1* (*R* = −0.12, *p* = 0.37), nor *RUNX1T1* (*R* = −0.2, *p* = 0.12) (Figure 2c).

Gene set enrichment analysis (GSEA) showed that the *TRH*-high group had significant activation of the oxidative phosphorylation pathway, citrate cycle, TCA cycle pathway, and the alanine aspartate and glutamate metabolism pathway, thus demonstrating the activation of metabolism-relevant pathways (Figure 2d and Figure S3a). In addition, we observed an activation of spliceosome and RNA polymerase pathways (Figure S3a). Further correlation analysis showed a significantly positive correlation between *TRH* and splicing factors *SRSF2* and *U2AF2* (Figure 2e). For the *TRH*-low group, GSEA exhibited an enrichment of the chemokine signaling pathway, cytokine–cytokine receptor interaction pathway, and the Fc gamma receptor-mediated phagocytosis pathway, indicating the activation of immune-related pathways (Figure S3b).

### 3.3. Clinical Characteristics of TRH Expression in the TCGA AML Dataset

**Figure 3:**
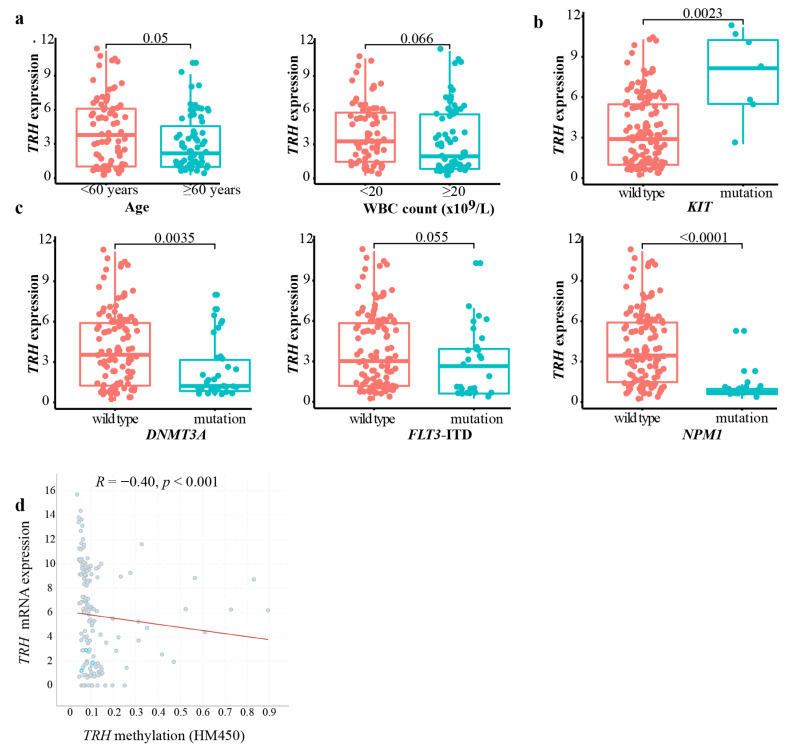
**Clinical and genetic correlation with *TRH* expression in the TCGA AML dataset.** (**a**) Comparison of *TRH* expression in different clinical subgroups of the TCGA AML dataset. (**b**) Comparison of *TRH* expression in the *KIT*–wild-type and *KIT*–mutation groups of TCGA AML. (**c**) Comparison of *TRH* expression in wild-type and mutation groups of *DNMT3A*, *FLT3-ITD,* and *NPM1* in the TCGA AML cohort. (**d**) Correlation of methylation status (HM450) with mRNA expression of *TRH* in TCGA AML using cBioportal. Spearman’s correlation analysis and correlation coefficient (*R*) are shown.

We further explored the clinical and genetic features of *TRH* expression in AML patients. With data from the TCGA AML project (*n* = 145), we found that AML patients older than 60 years had a lower expression of *TRH* (*p* = 0.05) (Figure 3a). There was no difference of *TRH* expression in either peripheral white blood count (WBC) or gender subgroups (Figure 3a and Figure S4).

We then analyzed the correlation of *TRH* expression with common driver mutations of AML. In the TCGA AML cohort, AML patients with *KIT* mutations and *RUNX1* mutations had a significantly higher expression levels of *TRH* compared with the wild-type groups (*p* = 0.0023 and *p* = 0.047, respectively) (Figure 3b and Figure S4). On the other hand, AML patients with the *DNMT3A* mutations and *NPM1* mutations presented significantly lower expression of *TRH* (*p* = 0.0035 and *p* < 0.001, respectively) compared with the corresponding wild-type groups (Figure 3c). However, we did not observe a significant difference of *TRH* expression in terms of *FLT3*-ITD mutation, *IDH1* mutation, *IDH2* mutation, or *TP53* mutation in TCGA AML cohort (Figure 3c and Figure S4). We also analyzed the effect of methylation status on *TRH* expression (Figure 3d) and found that there was a significant negative correlation between methylation and the *TRH* expression (*R* = −0.40, *p* < 0.001).

### 3.4. Validation of the Clinical Relevance of TRH Expression in Other AML Cohorts

**Figure 4:**
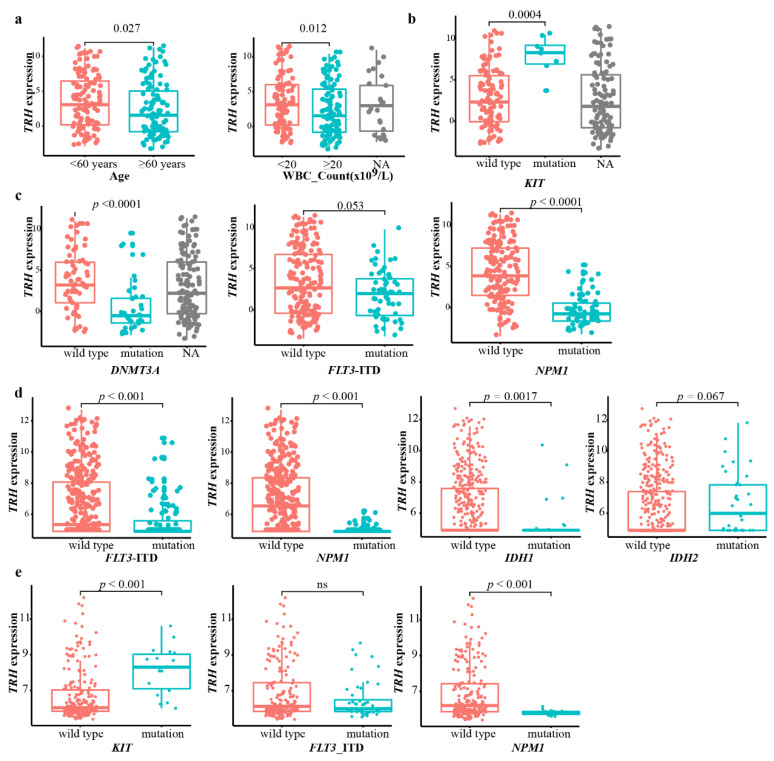
**Clinical and genetic correlation with *TRH* expression in Beat AML, GSE6891, and GSE17855 datasets**. (**a**) Comparison of *TRH* expression in different clinical subgroups in the Beat AML dataset. (**b**) Comparison of *TRH* expression in *KIT*–wild type and *KIT*–mutation groups from Beta AML. (**c**) Comparison of *TRH* expression in wild-type and mutation groups of *DNMT3A*, *FLT3*-ITD, and *NPM1* in Beat AML. (**d**) Comparison of *TRH* expression in different genetic subgroups of the GSE6891 dataset. (**e**) Comparison of *TRH* expression in different genetic subgroups of the GSE17855 dataset. Statistical significance was determined using the Wilcoxon test.

To validate the clinical and genetic relevance of *TRH* expression in other AML cohorts, we screened and obtained RNA-seq data of primary AML from Beat AML cohort (*n* = 223) [14]. We also observed a lower expression of *TRH* in AML patients older than 60 years old, reaching a statistical significance (*p* = 0.027) (Figure 4a). AML patients with a higher peripheral WBC had a significantly lower expression of *TRH* (*p* = 0.012) (Figure 4a). The genetic mutation pattern of the *TRH* expression in the Beat AML cohort was also analyzed. AML patients with *KIT* mutations had higher expression of *TRH* (*p* = 0.0004) (Figure 4b). In addition, patients with *DNMT3A* mutations and *NPM1* mutations had lower expression levels of *TRH* (*p* < 0.0001) (Figure 4c). Moreover, patients with the *KIT* mutation had a higher expression of *TRH* (*p* = 0.0004) (Figure 4b). These results in the Beat AML cohort were consistent with the TCGA AML cohort. Nevertheless, we did not observe a correlation between *TRH* expression and *RUNX1* mutation (Figure S5).

In addition, we also obtained gene expression data from GSE6891 (*n* = 461) [15,16] and GSE17855 (*n* = 237) [17,18,19]. With the available information of targeted gene mutations, we found that AML patients with *NPM1* mutations had significantly lower expression of *TRH* (*p* < 0.001) in both GSE6891 and GSE17855 (Figure 4d,e). In addition, AML patients with *FLT3*-ITD mutations had significantly lower expression of *TRH* in GSE6891 (*p* < 0.001) (Figure 4d). AML patients with the *KIT* mutation had a higher expression of *TRH* in GSE17855 (*p* < 0.001) (Figure 4e).

### 3.5. Immune Status and Drug Resistance Correlation with TRH Expression in AML

**Figure 5:**
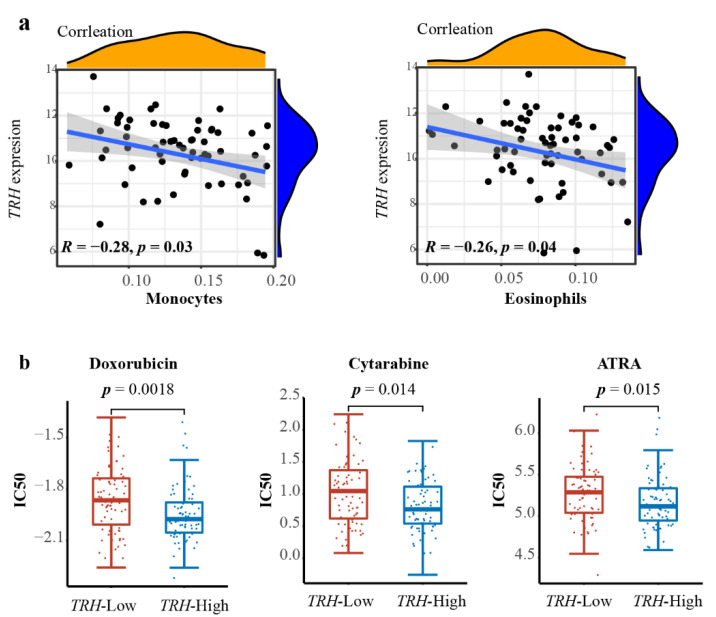
**Immune status and drug resistance correlated with *TRH* expression**. (**a**) Correlation analysis of *TRH* expression with the proportion of immune cells in *RUNX1-RUNX1T1* AML patients based on the Cibersort algorithm. Spearman’s correlation analysis and correlation coefficient (*R*) are shown. (**b**) Distribution of IC50 for doxorubicin, cytarabine, and all-trans retinoic acid (ATRA) between high- and low-*TRH* expression groups of AML patients. Statistical significance was determined using the Wilcoxon test.

Considering the activation of immune-related pathways in the *TRH*-low group, we then analyzed the immune infiltration in the bone marrow of *RUNX1-RUNX1T1* AML patients using the CIBERSORT algorithm [22]. Correlation analysis showed that there was a significant negative correlation between *TRH* expression and monocytes (*R* = −0.28, *p* = 0.03) and eosinophils (*R* = −0.26, *p* = 0.04) (Figure 5a). In the TCGA AML cohort, the CIBERSORT results of bone marrow of AML bone marrow showed that the *TRH* expression had a significant correlation with a variety of immune cells, including B cells, monocytes, neutrophils, NK cells and CD4 T cells (Figure S6). Consistent with the result in *RUNX1-RUNX1T1* AML, we observed a significant negative correlation of *TRH* expression with monocytes (*R* = −0.18, *p* = 0.03) (Figure S6).

Drug resistance is one of the leading reasons contributing to the treatment failure of AML patients. To test the effect of *TRH* expression on drug resistance, data from the GDSC database [28] was analyzed. We found that the *TRH*-high group had a significantly lower IC50 for AML chemotherapeutics, including cytarabine (*p* = 0.014) and doxorubicin (*p* = 0.0018) (Figure 5b), which indicated greater sensitivity to chemotherapy. We also observed that the *TRH*-high group had a lower IC50 for ATRA (*p* = 0.015), which was used in acute promyelocytic leukemia patients (Figure 5b).

### 3.6. Improvement of Prognostic Stratification for the ELN Risk System

**Figure 6:**
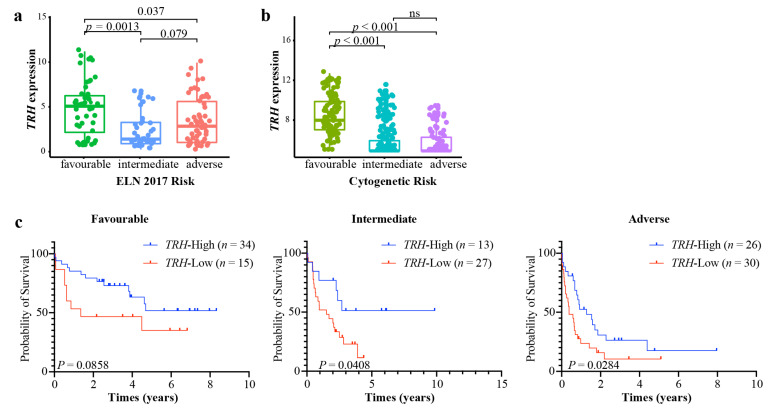
**Improvement of the ELN 2017 risk system with *TRH* expression**. (**a**) Comparison of *TRH* expression in different cytogenetic subgroups of TCGA AML according to the ELN 2017 risk system. (**b**) Comparison of *TRH* expression in different cytogenetic subgroups of GSE6891 dataset. Statistical significance was determined using the Wilcoxon test. (**c**) Kaplan–Meier plots of patients with high- and low-*TRH* expression in different risk groups of TCGA AML. Log-rank tests were used to compare the differences.

The European Leukemia Net (ELN) risk stratification system for AML [6] is commonly used. We further compared the *TRH* expression in different cytogenetic risk groups. In the TCGA AML cohort, we found that the favorable group of the ELN 2017 risk system had a significantly higher level of *TRH* expression than both the intermediate and adverse groups (Figure 6a). In the GSE6891 dataset, we also found that the good-risk group had a significantly higher expression of *TRH* (*p* < 0.001) (Figure 6b). Kaplan–Meier plots showed a significant survival difference between the high- and low-*TRH* groups in the intermediate group (*p* = 0.0408) and adverse group (*p* = 0.284), respectively, in the TCGA AML cohort (Figure 6c). Furthermore, univariate and multivariate analyses were performed to assess whether *TRH* expression had prognostic significance (Table 1). The results of univariate analysis showed the association of OS with age (HR, 1.040; 95%CI, 1.025–1.056; *p* < 0.001), WBC count (HR, 1.005; 95%CI, 1.001–1.009; *p* = 0.016), *TRH* expression (HR, 0.868; 95%CI, 0.800–0.940; *p* = 0.001), and ELN 2017 risk (HR, 1.865; 95%CI, 1.445–2.407; *p* < 0.001). The multivariate analysis showed that age (HR, 1.035; 95%CI, 1.019–1.051; *p* < 0.001), WBC count (HR, 1.007; 95%CI, 1.002–1.011; *p* = 0.008), *TRH* expression (HR, 0.910; 95%CI, 0.840–0.987; *p* = 0.022), and ELN 2017 risk (HR, 1.655; 95%CI, 1.254–2.185; *p* < 0.001) remained independent prognostic factors for OS. Collectively, these data indicate that the expression of *TRH* could be used to further stratify AML patients on the basis of the ELN 2017 risk system.

## 4. Discussion

In this work, based on what we discovered previously in *RUNX1-RUNX1T1* AML, we further extended the research of *TRH* expression in AML. We revealed the genetic, immunological, and clinical features correlated with *TRH* expression in the TCGA AML cohort, and further validated these results in three other cohorts. Furthermore, we showed that *TRH* expression could complement and refine the accuracy of the ELN 2017 risk stratification system for AML.

*TRH* is known to be a component of the hypothalamic–pituitary–thyroid axis, and is involved in the regulation and release of thyroid-stimulating hormone. Recently, with single-cell RNA-seq, we revealed heterogeneous myeloblast populations and their relevant biomarkers in patients with *RUNX1-RUNX1T1* AML [8,9]. *TRH*, as one of the relevant biomarkers, was proved to be an independent prognostic risk factor in *RUNX1-RUNX1T1* AML. In this study, we found that the top activated pathways in the *TRH*-high group of *RUNX1-RUNX1T1* AML were the oxidative phosphorylation pathway, the citrate cycle, the TCA cycle pathway, and the alanine aspartate and glutamate metabolism pathway. This revealed the aberrant activation of metabolism signaling pathways in the *TRH*-high group. In addition, the expression of *TRH* in t (8;21) AML was not regulated by the *RUNX1*-*RUNX1T1* fusion. This might be due to genetic or epigenetic changes leading to the regulation of *TRH* expression. In fact, through the query of the RNA-seq data of pan cancer, *TRH* was barely expressed in a variety of cancers, which might be attributed to its methylation status [30,31].

With data from the MILE study [15,25], we analyzed and compared *TRH* expression in a variety of hematological cancers including ALL, CLL, CML, and MDS. Unlike AML patients, those with these hematological diseases presented relatively lower expression levels of *TRH*, suggesting that *TRH* was a characteristic biomarker for AML. We further explored the underlying correlation of *TRH* expression with genetic changes in AML. We observed lower expression levels of *TRH* when AML patients harbored *DNMT3A* or *NPM1* mutations in four independent cohorts. However, when AML patients carried *KIT* mutations, they presented higher *TRH* expression levels. This may be due to the fact that the *KIT* mutation frequently occurred in patients with *RUNX1-RUNX1T1* AML. In addition, *TRH* was reported to be highly expressed in *RUNX1-RUNX1T1* AML patients by other studies [32,33].

Early identification of high-risk AML and administration of intensive chemotherapy and transplantation could greatly reduce the relapse rate. Thus, the European Leukemia Net has proposed a risk stratification system that incorporates cytogenetic mutational status and has been widely used clinically [6]. Recently, a novel AML prognostic score (APS) was proposed and demonstrated that RNA-seq might have additional advantages for clinical assessment [7]. In this study, we showed that different risk groups of the ELN 2017 risk system had differential expression of *TRH*, which could be used to further stratify AML patients. We also found that patients with higher expression of *TRH* in AML appeared to be more sensitive to chemotherapy. Future studies are still needed to explore the drugs that were more effective in the *TRH*-low group.

In conclusion, we explored the clinical and genetic features of a novel biomarker, *TRH*, in AML patients. Our preliminary analysis suggests that *TRH* could refine the ELN 2017 risk stratification system.

## Figures and Tables

**Table 1:** Uni- and multi-variate Cox regression analysis for overall survival of TCGA AML.

Factors	Univariate		Multivariate	
	HR (95%CI)	*p*-Value	HR (95%CI)	*p*-Value
Age (years)	1.040 (1.025–1.056)	< 0.001	1.035 (1.019–1.051)	< 0.001
Gender (male vs. female)	0.989 (0.658–1.485)	0.956		
BM blasts (%)	0.999 (0.989–1.009)	0.827		
PB blasts (%)	1.003 (0.997–1.010)	0.356		
WBC count	1.005 (1.001–1.009)	0.016	1.007 (1.002–1.011)	0.008
*TRH* expression	0.868 (0.800–0.940)	0.001	0.910 (0.840–0.987)	0.022
ELN 2017 risk	1.865 (1.445–2.407)	< 0.001	1.655 (1.254–2.185)	< 0.001

## Data Availability

Publicly available datasets including TCGA, Beat AML, GSE6891, and GSE17855 can be downloaded from online. Data of AML with *RUNX1*-*RUNX1T1* can be accessed on the National Omics Data Encyclopedia (http://www.biosino.org/node/project/detail/OEP000629 (accessed on 12 December 2021)).

## Supplementary Materials

The following supporting information can be downloaded at: https://www.mdpi.com/article/10.3390/biom12101359/s1. Figure S1. Expression of *TRH* in hematological cancer and normal tissues. (a) Expression of *TRH* in hematological cancer with data from MILE study via BloodSpot. (b) t-distributed stochastic neighbor embedding (tSNE) plots displaying the expression of *TRH* from samples of both fetal and adult tissue; Figure S2. Kaplan–Meier plots of *TRH* expression in ovarian serous cystadenocarcinoma (OV) and subgroup of AML in the TCGA project. Kaplan–Meier plots of disease-free survival (a) and overall survival (b) between high- and low-*TRH* groups in OV. Subgroup analysis (c–e) of high- and low-*TRH* groups in the TCGA AML project based on the FAB category; Figure S3. Gene set enrichment analysis (GSEA) of *TRH* expression in t (8;21) AML patients. (a) Top enriched pathways in the high-*TRH* (*n* = 31) group classified according to the median level of *TRH* expression from the 62 de novo t (8;21) AML patients. (b) Top enriched pathways in the low-*TRH* (*n* = 31) group classified according to the median level of *TRH* expression from the 62 de novo t (8;21) AML patients; Figure S4. Clinical and genetic correlation with *TRH* expression in TCGA AML datasets. Statistical significance was determined using the Wilcoxon test; Figure S5. Clinical and genetic correlation with *TRH* expression in the Beat AML datasets. Statistical significance was determined using the Wilcoxon test; Figure S6. Correlation of *TRH* expression with the proportion of immune cells in TCGA AML datasets. Spearman’s correlation analysis and correlation coefficient (*R*) are shown.

## Abbreviations

ACC (adrenocortical carcinoma), BLCA (bladder urothelial carcinoma), BRCA (breast invasive carcinoma), CESC (cervical squamous cell carcinoma and endocervical adenocarcinoma), CHOL (cholangiocarcinoma), COAD (colon adenocarcinoma), DLBC (lymphoid neoplasm diffuse large B-cell lymphoma), ESCA (esophageal carcinoma), GBM (glioblastoma multiforme), HNSC (head and neck squamous cell carcinoma), KICH (kidney chromophobe), KIRC (kidney renal clear cell carcinoma), KIRP (kidney renal papillary cell carcinoma), LAML (acute myeloid leukemia), LGG (brain lower grade glioma), LIHC (liver hepatocellular carcinoma), LUAD (lung adenocarcinoma), LUSC (lung squamous cell carcinoma), OV (ovarian serous cystadenocarcinoma), PAAD (pancreatic adenocarcinoma), PCPG (pheochromocytoma and paraganglioma), PRAD (prostate adenocarcinoma), READ (rectum adenocarcinoma), SARC (sarcoma), SKCM (skin cutaneous melanoma), STAD (stomach adenocarcinoma), TGCT (testicular germ cell tumors), THCA (thyroid carcinoma), THYM (thymoma), UCEC (uterine corpus endometrial carcinoma), UCS (uterine carcinosarcoma).

## References

[1] Papaemmanuil E., Gerstung M., Bullinger L., Gaidzik V.I., Paschka P., Roberts N.D., Potter N.E., Heuser M., Thol F., Bolli N. (2016). Genomic Classification and Prognosis in Acute Myeloid Leukemia. N. Engl. J. Med..

[2] Juliusson G., Antunovic P., Derolf Å., Lehmann S., Möllgård L., Stockelberg D., Tidefelt U., Wahlin A., Hoglund M. (2009). Age and acute myeloid leukemia: Real world data on decision to treat and outcomes from the Swedish Acute Leukemia Registry. Blood.

[3] Döhner H., Weisdorf D.J., Bloomfield C.D. (2015). Acute Myeloid Leukemia. N. Engl. J. Med..

[4] Shallis R.M., Wang R., Davidoff A., Ma X., Zeidan A.M. (2019). Epidemiology of acute myeloid leukemia: Recent progress and enduring challenges. Blood Rev..

[5] The Cancer Genome Atlas Research Network (2013). Genomic and epigenomic landscapes of adult de novo acute myeloid leukemia. N. Engl. J. Med..

[6] Döhner H., Estey E., Grimwade D., Amadori S., Appelbaum F.R., Büchner T., Dombret H., Ebert B.L., Fenaux P., Larson R.A. (2017). Diagnosis and management of AML in adults: 2017 ELN recommendations from an international expert panel. Blood.

[7] Docking T.R., Parker J.D.K., Jädersten M., Duns G., Chang L., Jiang J., Pilsworth J.A., Swanson L.A., Chan S.K., Chiu R. (2021). A clinical transcriptome approach to patient stratification and therapy selection in acute myeloid leukemia. Nat. Commun..

[8] Jiang L., Li X.-P., Dai Y.-T., Chen B., Weng X.-Q., Xiong S.-M., Zhang M., Huang J.-Y., Chen Z., Chen S.-J. (2020). Multidimensional study of the heterogeneity of leukemia cells in t(8;21) acute myelogenous leukemia identifies the subtype with poor outcome. Proc. Natl. Acad. Sci. USA.

[9] Li X., Dai Y., Chen B., Huang J., Chen S., Jiang L. (2021). Clinical significance of CD34+CD117dim/CD34+CD117bri myeloblast-associated gene expression in t(8;21) acute myeloid leukemia. Front. Med..

[10] Gary K.A., Sevarino K.A., Yarbrough G.G., Prange A.J., Winokur A., Lee M.C., Smith F.L., Stevens D.L., Welch S.P. (2003). The Thyrotropin-Releasing Hormone (TRH) Hypothesis of Homeostatic Regulation: Implications for TRH-Based Therapeutics. J. Pharmacol. Exp. Ther..

[11] Yarbrough G., Kamath J., Winokur A., Prange A. (2007). Thyrotropin-releasing hormone (TRH) in the neuroaxis: Therapeutic effects reflect physiological functions and molecular actions. Med. Hypotheses.

[12] Mullur R., Liu Y.-Y., Brent G.A. (2014). Thyroid Hormone Regulation of Metabolism. Physiol. Rev..

[13] Ribeiro M.O. (2008). Effects of Thyroid Hormone Analogs on Lipid Metabolism and Thermogenesis. Thyroid.

[14] Tyner J.W., Tognon C.E., Bottomly D., Wilmot B., Kurtz S.E., Savage S.L., Long N., Schultz A.R., Traer E., Abel M. (2018). Functional genomic landscape of acute myeloid leukaemia. Nat. Cell Biol..

[15] Verhaak R.G., Wouters B.J., Erpelinck C.A., Abbas S., Beverloo H.B., Lugthart S., Lowenberg B., Delwel R., Valk P.J. (2009). Prediction of molecular subtypes in acute myeloid leukemia based on gene expression profiling. Haematologica.

[16] de Jonge H.J.M., Valk P.J.M., Veeger N.J.G.M., ter Elst A., Boer M.L.D., Cloos J., de Haas V., Heuvel-Eibrink M.M.V.D., Kaspers G.J.L., Zwaan C.M. (2010). High VEGFC expression is associated with unique gene expression profiles and predicts adverse prognosis in pediatric and adult acute myeloid leukemia. Blood.

[17] Balgobind B.V., Van den Heuvel-Eibrink M.M., De Menezes R.X., Reinhardt D., Hollink I.H.I.M., Arentsen-Peters S.T.J.C.M., van Wering E.R., Kaspers G.J., Cloos J., de Bont E.S. (2011). Evaluation of gene expression signatures predictive of cytogenetic and molecular subtypes of pediatric acute myeloid leukemia. Haematologica.

[18] Sandahl J.D., Coenen E.A., Forestier E., Harbott J., Johansson B., Kerndrup G., Adachi S., Auvrignon A., Beverloo H.B., Cayuela J.-M. (2014). t(6;9)(p22;q34)/DEK-NUP214-rearranged pediatric myeloid leukemia: An international study of 62 patients. Haematologica.

[19] Hartsink-Segers S., Zwaan C., Exalto C., Luijendijk M.W.J., Calvert V., Petricoin E.F., Evans W., Reinhardt D., De Haas V., Hedtjärn M. (2012). Aurora kinases in childhood acute leukemia: The promise of aurora B as therapeutic target. Leukemia.

[20] Love M.I., Huber W., Anders S. (2014). Moderated estimation of fold change and dispersion for RNA-seq data with DESeq2. Genome Biol..

[21] Patro R., Duggal G., Love M.I., Irizarry R.A., Kingsford C. (2017). Salmon provides fast and bias-aware quantification of transcript expression. Nat. Methods.

[22] Newman A.M., Liu C.L., Green M.R., Gentles A.J., Feng W., Xu Y., Hoang C.D., Diehn M., Alizadeh A.A. (2015). Robust enumeration of cell subsets from tissue expression profiles. Nat. Methods.

[23] Tang Z., Kang B., Li C., Chen T., Zhang Z. (2019). GEPIA2: An enhanced web server for large-scale expression profiling and interactive analysis. Nucleic Acids Res..

[24] Bagger F.O., Kinalis S., Rapin N. (2018). BloodSpot: A database of healthy and malignant haematopoiesis updated with purified and single cell mRNA sequencing profiles. Nucleic Acids Res..

[25] Haferlach T., Kohlmann A., Wieczorek L., Basso G., Kronnie G.T., Béné M.-C., De Vos J., Hernández J.M., Hofmann W.-K., Mills K.I. (2010). Clinical Utility of Microarray-Based Gene Expression Profiling in the Diagnosis and Subclassification of Leukemia: Report From the International Microarray Innovations in Leukemia Study Group. J. Clin. Oncol..

[26] Zheng R., Wan C., Mei S., Qin Q., Wu Q., Sun H., Chen C.-H., Brown M., Zhang X., A Meyer C. (2018). Cistrome Data Browser: Expanded datasets and new tools for gene regulatory analysis. Nucleic Acids Res..

[27] Gao J., Aksoy B.A., Dogrusoz U., Dresdner G., Gross B.E., Sumer S.O., Sun Y., Jacobsen A., Sinha R., Larsson E. (2013). Integrative Analysis of Complex Cancer Genomics and Clinical Profiles Using the cBioPortal. Sci. Signal..

[28] Yang W., Soares J., Greninger P., Edelman E.J., Lightfoot H., Forbes S., Bindal N., Beare D., Smith J.A., Thompson I.R. (2012). Genomics of Drug Sensitivity in Cancer (GDSC): A resource for therapeutic biomarker discovery in cancer cells. Nucleic Acids Res..

[29] Han X., Zhou Z., Fei L., Sun H., Wang R., Chen Y., Chen H., Wang J., Tang H., Ge W. (2020). Construction of a human cell landscape at single-cell level. Nature.

[30] Shimizu H., Horii A., Sunamura M., Motoi F., Egawa S., Unno M., Fukushige S. (2011). Identification of epigenetically silenced genes in human pancreatic cancer by a novel method “microarray coupled with methyl-CpG targeted transcriptional activation” (MeTA-array). Biochem. Biophys. Res. Commun..

[31] Arai E., Chiku S., Mori T., Gotoh M., Nakagawa T., Fujimoto H., Kanai Y. (2012). Single-CpG-resolution methylome analysis identifies clinicopathologically aggressive CpG island methylator phenotype clear cell renal cell carcinomas. Carcinogenesis.

[32] Bullinger L., Rücker F.G., Kurz S., Du J., Scholl C., Sander S., Corbacioglu A., Lottaz C., Krauter J., Fröhling S. (2007). Gene-expression profiling identifies distinct subclasses of core binding factor acute myeloid leukemia. Blood.

[33] Hsu C.-H., Nguyen C., Yan C., Ries R.E., Chen Q.-R., Hu Y., Ostronoff F., Stirewalt D.L., Komatsoulis G., Levy S. (2015). Transcriptome Profiling of Pediatric Core Binding Factor AML. PLoS ONE.

