# Supplementary figures and images for: Identification of the Thyrotropin-Releasing Hormone (TRH) as a Novel Biomarker in the Prognosis for Acute Myeloid Leukemia
Source: Biomolecules. 2022 Sep 23;12(10):1359. doi: 10.3390/biom12101359 (PMC9599642; doi:10.3390/biom12101359)

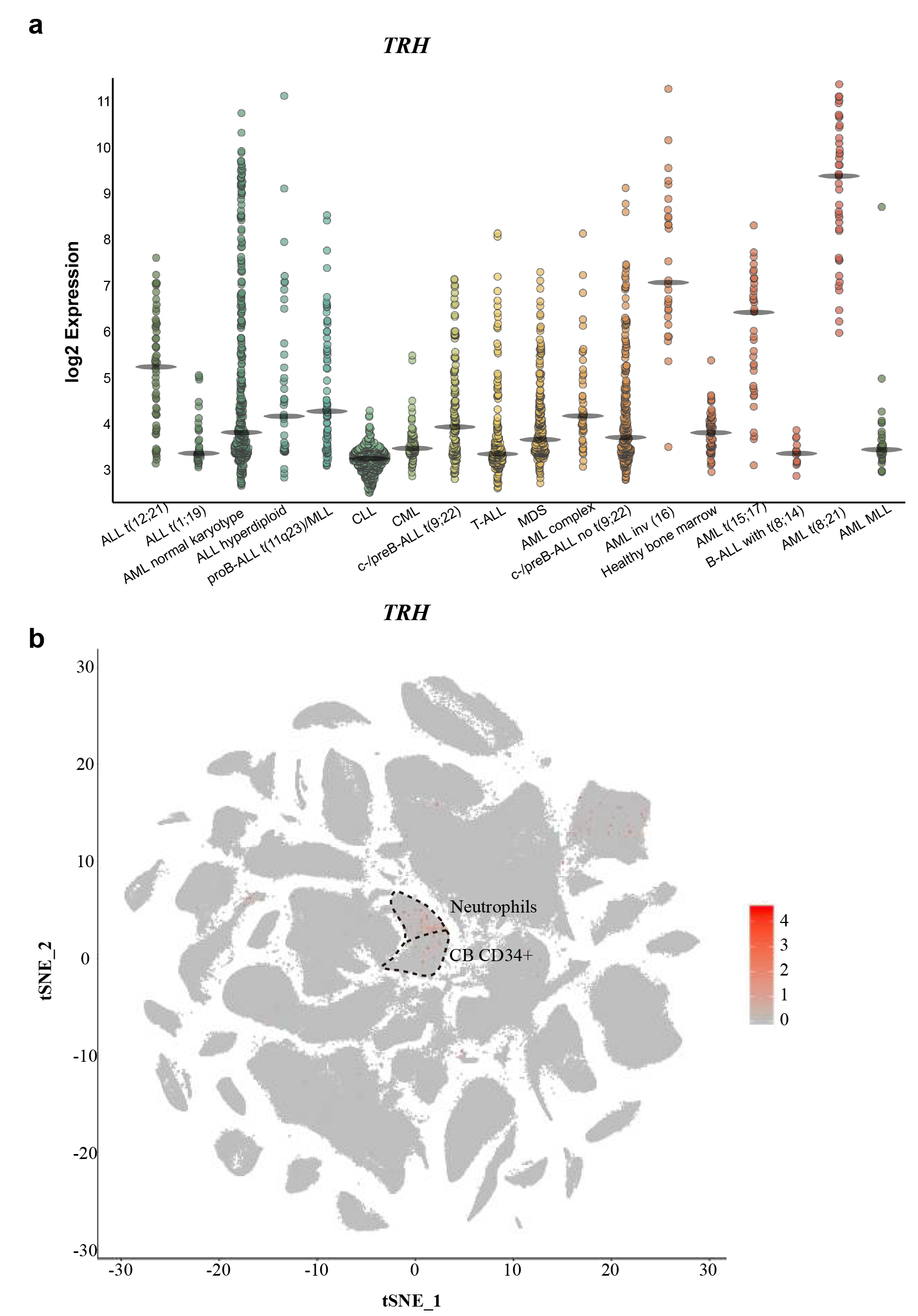

Supplement: Supplementary file 1 [file biomolecules-12-01359-s001.zip › biomolecules-1916807-supplementary/biomolecules-1916807-supplementary re 1 new/Figure S1.tif]

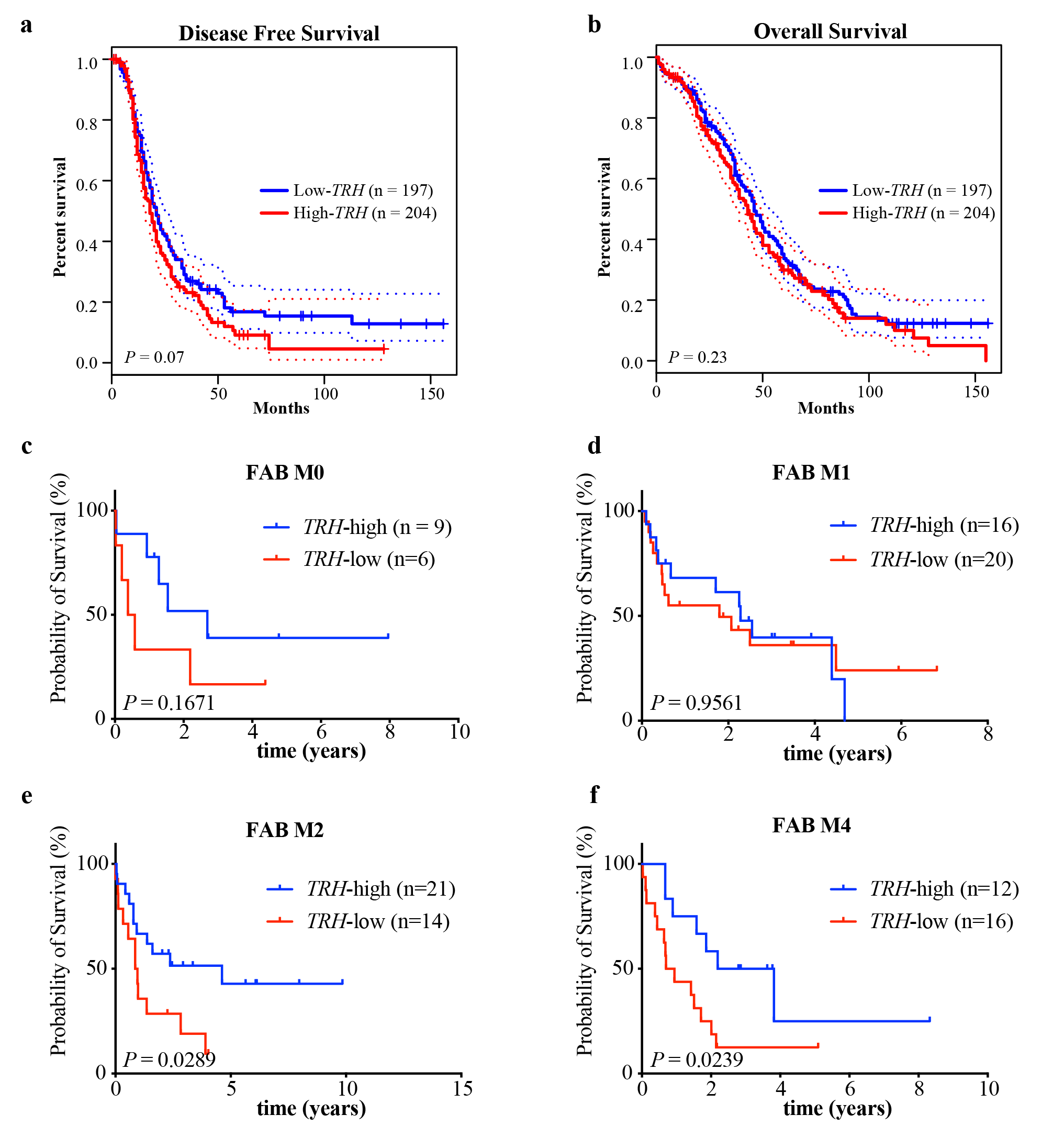

Supplement: Supplementary file 1 [file biomolecules-12-01359-s001.zip › biomolecules-1916807-supplementary/biomolecules-1916807-supplementary re 1 new/Figure S2.tif]

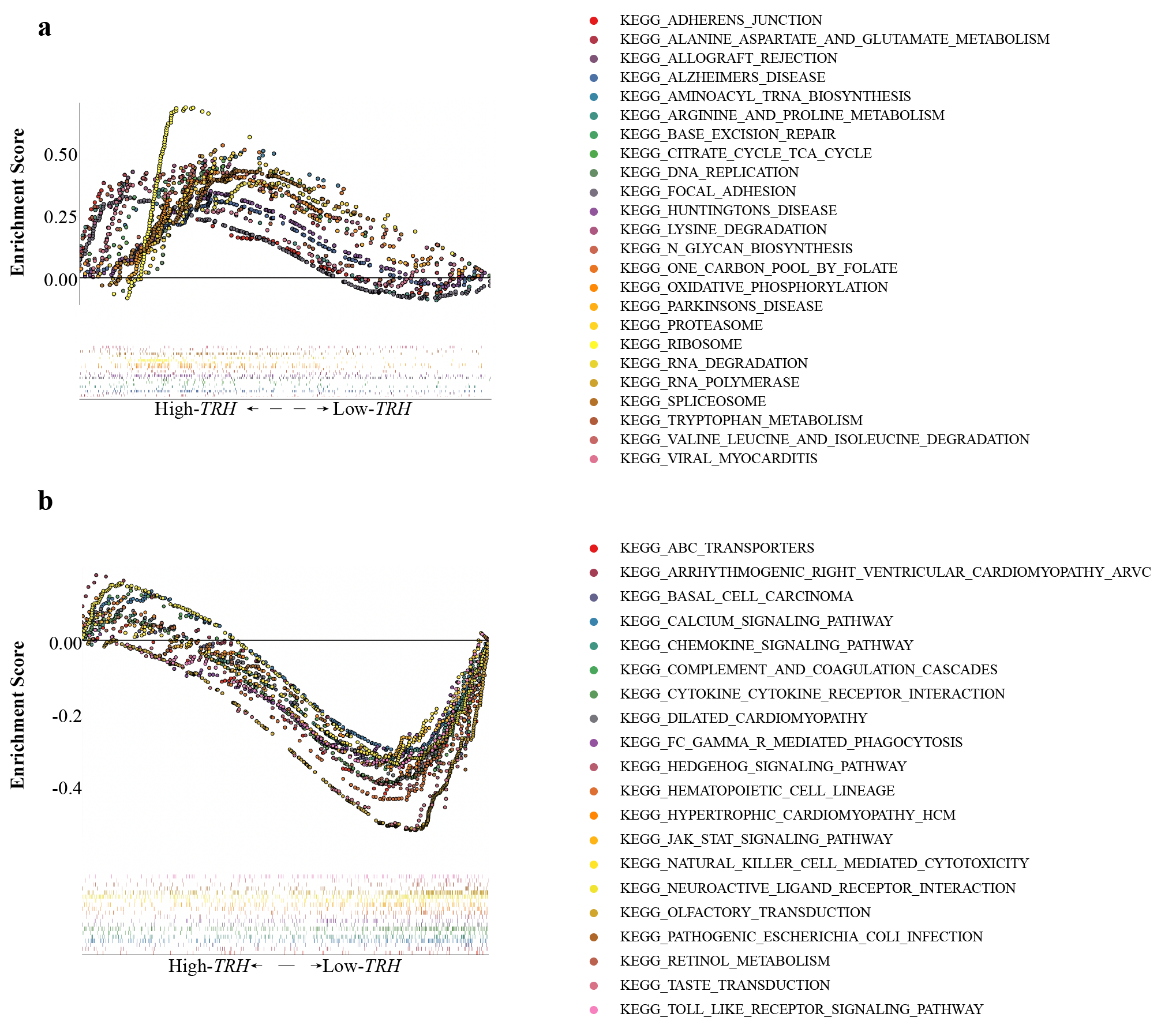

Supplement: Supplementary file 1 [file biomolecules-12-01359-s001.zip › biomolecules-1916807-supplementary/biomolecules-1916807-supplementary re 1 new/Figure S3.tif]

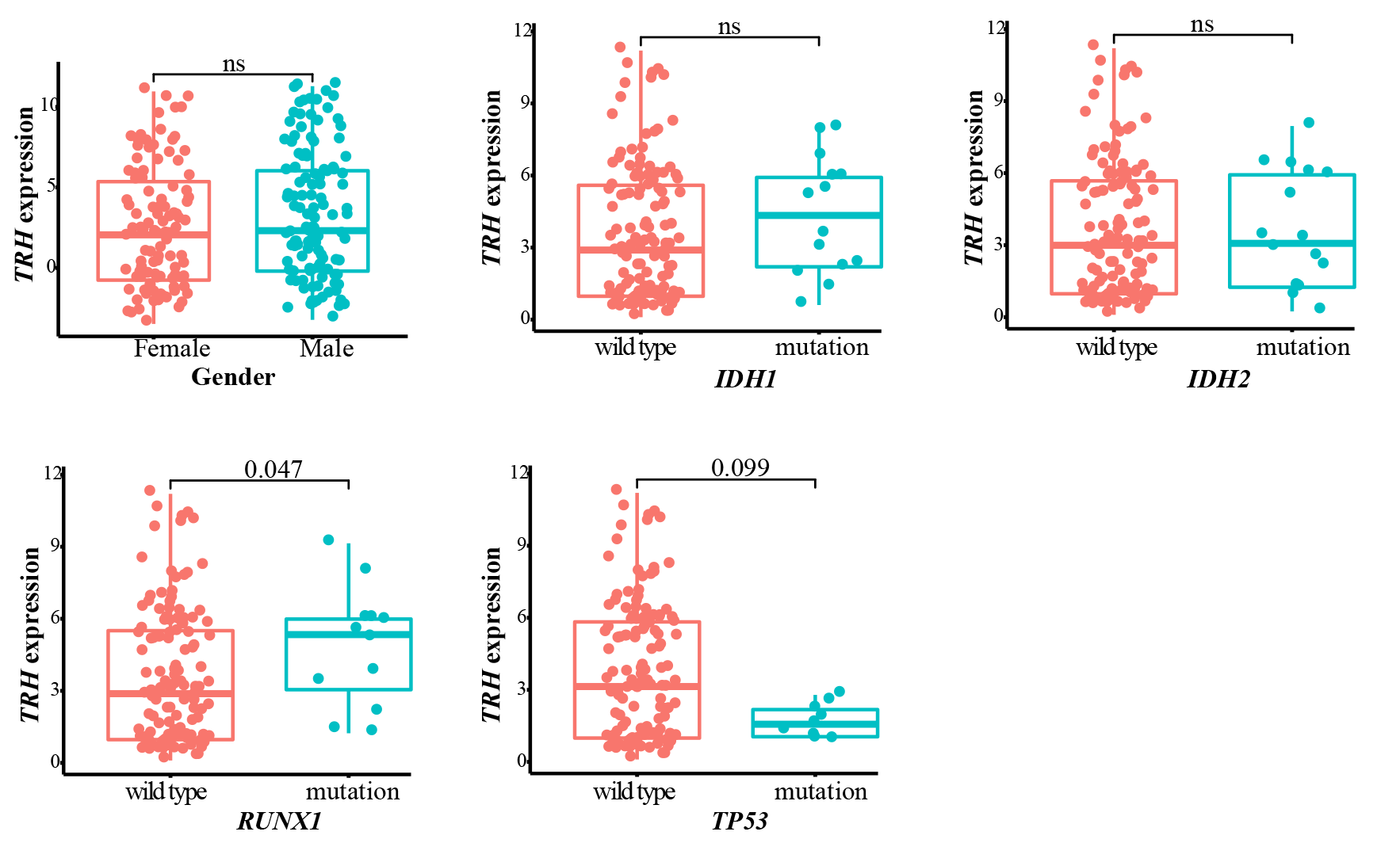

Supplement: Supplementary file 1 [file biomolecules-12-01359-s001.zip › biomolecules-1916807-supplementary/biomolecules-1916807-supplementary re 1 new/Figure S4.tif]

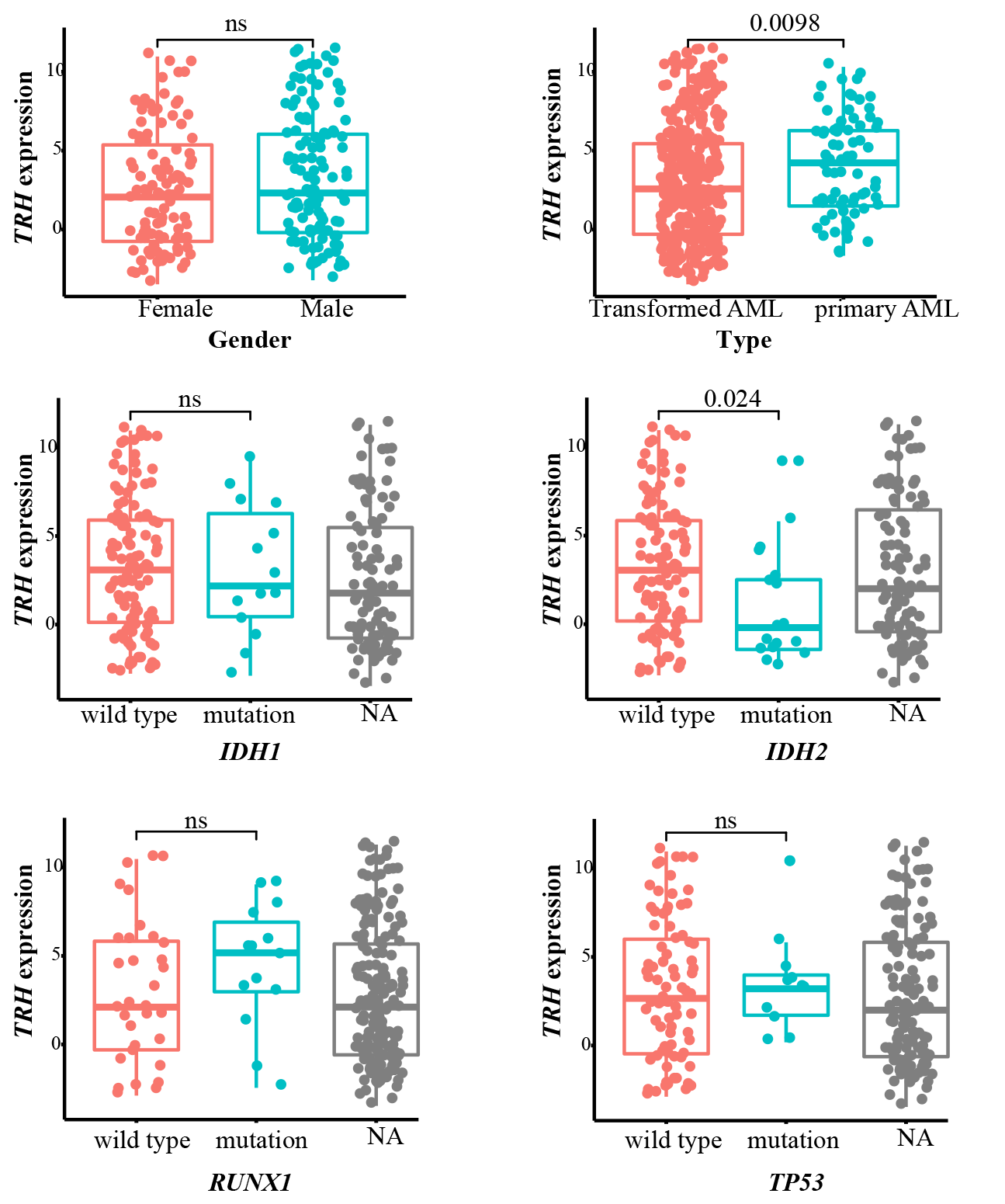

Supplement: Supplementary file 1 [file biomolecules-12-01359-s001.zip › biomolecules-1916807-supplementary/biomolecules-1916807-supplementary re 1 new/Figure S5.tif]

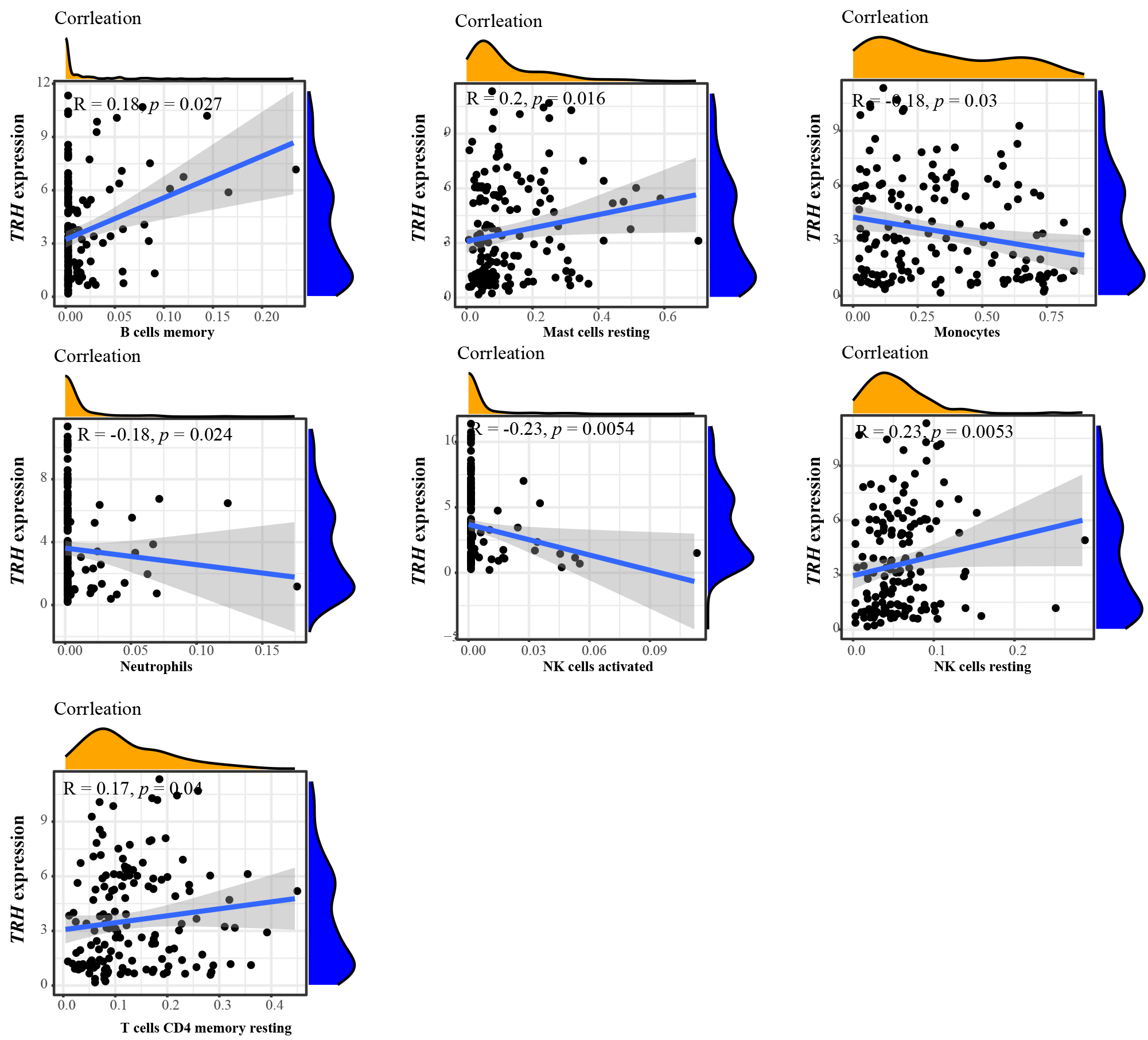

Supplement: Supplementary file 1 [file biomolecules-12-01359-s001.zip › biomolecules-1916807-supplementary/biomolecules-1916807-supplementary re 1 new/Figure S6.tif]
